# Protocol of the evolution study: A prospective, multicenter, observational study evaluating the effect and health economics of endovascular treatment in patients with moderate and severe calcification of femoropopliteal artery

**DOI:** 10.3389/fcvm.2022.1039313

**Published:** 2022-10-18

**Authors:** Jun Pan, Lianrui Guo, Xin Fang, Zibo Feng, Qiang Li, Chunshui He, Hongfei Sang, Weihao Shi, Zhenyu Shi, Bing Wang, Chenyang Qiu, Ziheng Wu, Meng Ye

**Affiliations:** ^1^Department of Vascular Surgery, The First Affiliated Hospital, School of Medicine, Zhejiang University, Hangzhou, China; ^2^Department of Vascular Surgery, Xuanwu Hospital Capital Medical University, Beijing, China; ^3^Department of Vascular Surgery, Affiliated Hangzhou First People's Hospital, School of Medicine, Zhejiang University, Hangzhou, China; ^4^Department of Vascular Surgery, Liyuan Hospital Affiliated Tongji Medical College of Huazhong University of Science and Technology, Wuhan, China; ^5^Department of Vascular Surgery, Qingdao Haici Hospital Affiliated to Qingdao University, Qingdao, China; ^6^Department of Vascular Surgery, Hospital of Chengdu University of Traditional Chinese Medicine, Chengdu, China; ^7^Department of Vascular Surgery, The Second Affiliated Hospital of Soochow University, Suzhou, China; ^8^Department of Vascular Surgery, School of Medicine, Huashan Hospital, Fudan University, Shanghai, China; ^9^Department of Vascular Surgery, School of Medicine, Zhongshan Hospital, Fudan University, Shanghai, China; ^10^Department of Vascular Surgery, School of Medicine, Renji Hospital, Shanghai Jiaotong University, Shanghai, China

**Keywords:** calcification, endovascular treatment, protocol, health economics, peripheral artery disease

## Abstract

**Objectives:**

Peripheral artery disease with calcification is extremely prevalent in the elderly. Due to the calcification, it requires a different clinical approach than the more common arteriosclerosis obliterans of the lower extremity. The introduction of novel technologies such as the drug-coated balloon, directional atherectomy, supera and drug-eluting stent has improved the prognosis of these patients. This study will contribute to the development of higher-quality evidence-based medicine for clinical treatment by assessing the quality of life (QOL), clinical treatment effect, and health economics of patients with calcification.

**Method and analysis:**

The Evolution study is designed as a prospective, multicenter, observational, real-world study. From January 2021 to December 2022, 600 patients with moderate to severe femoropopliteal artery calcification will be recruited from ten locations in China. After discharge, information on demographics, disease history, procedure details, imaging findings, and follow-up will be collected. Patients will undergo follow-up at 1, 6, 12, 18, and 24 months after operation. Technical success rate, vascular quality of life questionnaire, primary patency rate of the target lesion, clinically driven target lesion revascularization rate (CD-TLR), and health economics evaluation are all included as outcome measures.

**Conclusions:**

The Evolution study helps to investigate the clinical and financial results of various endovascular therapy modalities for patients with moderate and severe femoropopliteal artery calcification. These actual facts may help to harmonize therapy recommendations for peripheral artery disease.

**Clinical trial registration:**

The study protocol was registered at www.clinicaltrials.gov (registration number: NCT04716361).

## Introduction

Peripheral artery disease is a collection of chronic ischemic illnesses that result in arterial stenosis and even occlusion due to arterial intimal thickening, calcification, and secondary thrombosis (1, 2). It is a form of systemic arteriosclerosis that manifests locally. Frequently, the distal abdominal aorta, iliac, femoral popliteal, and other big and middle arteries are involved, with the distal popliteal artery and its branches being impacted later (2–4). Clinical signs range in severity from fear of cold to weariness after walking, intermittent claudication, resting pain, ulceration, and necrosis (5). Middle-aged and older adults are more likely to get this condition. With population aging and changes in diet structure, the incidence rate of this condition has steadily climbed, reaching ~15–30% among the 70 year old population, and has become one of the most common disorders encountered during vascular surgery (6–8).

Peripheral artery disease with calcification is a relatively prevalent complication in clinic, particularly in the elderly (9, 10). Due to the disease's calcification, it requires a different clinical approach than the more common peripheral artery disease (10, 11). Simple balloon dilatation and stent insertion have a dismal prognosis. The prognosis of such patients has improved with the emergence of new technologies such as drug-coated balloons, directional atherectomy, supera and drug-eluting stents (12–15). There are currently a few reports on these therapy regimens. While these studies demonstrate positive results, they are largely based on retrospective, single-center cohort studies (16, 17). Additionally, patients' quality of life scores and health economic evaluations are still relatively uninvolved. Therefore, this Evolution study will be conducted as a prospective, multicenter, observational, real-world study, focusing on evaluating the quality-of-life score (QOL), clinical treatment effect and health economics of patients with calcification. Our study will provide higher quality evidence-based medicine for clinical treatment.

## Materials and methods

### Study design

The Evolution is a multicenter, prospective, real-world observational clinical trial. It will take place between January 2021 and December 2022. The study included 600 individuals with moderate to severe femoropopliteal artery calcification. These patients will come from ten different hospitals in China. The freedom from TLR (target lesion revascularization), primary patency rate of the femoropopliteal artery, improvement in quality-of-life score, and health economics will all be monitored over the course of 2 years for each patient. The Department of Vascular Surgery, First Affiliated Hospital, School of Medicine, Zhejiang University developed and initiated the study.

### Patient and public involvement

Patients and the public were not involved in the design of this protocol.

### Participants

We will screen all patients who got endovascular therapy in the 10 approved centers in China between January 2021 and December 2022. A total of 600 individuals with moderate to severe femoropopliteal artery calcification will be recruited. All patients will be assessed for inclusion and exclusion criteria prior to enrollment in the research. Patients who do not meet these criteria will be excluded from the research. The recruitment process is non-competitive and will have no bearing on clinical practice.

## Criteria

### Inclusion criteria

- Rutherford category of 2–6.
- Patients with at least 90% stenosis of the moderate to severe calcification popliteal artery with the length of the continuous calcification on the affected side of ≥5 cm will be included (the segment length would be measured by ultrasound, CTA and DSA).
- If a patient requires treatment on both lower limbs, include the side with the most significant calcification. If both sides have a similar degree of calcification, choose the first treatment side.
- At the distal end of the knee, the patient should have an unobstructed outflow tract that is at least 1/3 the length of the patent pedal artery.
- Patients whose initial treatment failed due to the guide wire being unable to pass through the lesion may be included if subsequent endovascular interventional therapy is successful.
- Endovascular treatment is not limited in any way, and it may involve a variety of endovascular instruments and technologies that are currently recognized and employed in the real world, both at home and abroad.

### Exclusion criteria

- Life expectancy of patients is <12 months.
- Patients with severe infection, gangrene, or tissue defect beyond the toe plane (major tissue loss), who may require substantial amputation following endovascular treatment.
- The quality of patients' life cannot be assessed by vascular quality of life questionnaire due to difficulties in communication.
- Patients who have chronic femoropopliteal occlusive disease without calcification.
- Patients with acute arterial thrombosis.
- Limbs that have been treated with the femoral and popliteal artery bypass surgery.
- Pregnant and lactating women.
- Patients who have a history of hypersensitivity to contrast agents or drugs utilized in endovascular intervention.
- Patients who engage concurrently in clinical studies for other medicines or technologies.
- Patients who are diagnosed within 14 days of surgery with an active systemic infection or uncontrolled coagulopathy.
- Patients with severe diseases, such as liver failure, advanced tumor, severe cardiac dysfunction and abnormal coagulation caused by genetic diseases.
- Patients with myocardial infarction, unstable angina or cerebral infarction within the past 6 months.

### Endovascular procedure

Endovascular treatment will be used to treat all of the patients. The contralateral common femoral artery, ipsilateral common femoral artery, or brachial artery are used for vascular access. The retrograde popliteal artery puncture strategy will be used if the anterograde approach fails. The guide wire will then be put through the lesion, followed by angiography to determine the severity, length, and position of the calcification. Then, a variety of endovascular therapies will be used, depending on the lesion's features and the physician's preferences. For instance: drug-coated balloon angioplasty, drug-coated balloon angioplasty and stent implantation, directional atherectomy, drug-eluting stent, novel dedicated stent (Supera) or combination of above.

### Termination criteria

Subjects are free to exit the research at any time for any reason. This will have no impact on how patients are treated in the future. On the following circumstance, the investigator may elect to remove the participant from the research.

- The patient is lost to follow-up.
- The patient revokes his or her informed permission on his or her own.
- Serious violation of the study protocol by investigators or subjects.
- The subject is necessary to discontinue form the study for other reasons such as occurrence of more serious adverse events.

The relevant main and secondary outcomes would be deleted for participants who dropped out of the experiment.

### Recruitment

Clinical practice will be used to find suitable patients. All potential participants will have a standard checkup as well as an angiography examination to determine the severity of vascular lesions. After receiving informed permission, eligible patients will be enrolled.

### Data collection

At the start of this investigation, an uniform case report form (CRF) will be created. The demographics, medical history, imaging characteristics, surgery details, treatment results and complications, as well as follow-up information will all be collected using this CRF. The data entry is done separately by two investigators, and the data input verification is done to reduce the likelihood of error. Any incorrect data or words should be highlighted with a single line and filled in with the proper data or words on the side, along with the investigator's signature and the current date. The original CRF will be stored by the sponsor, while a copy will be retained at the center and converted to electronic records in the associated data management system. The lower extremity artery data management system, which is based on the concept of real-world research, is used to gather data for this study. Preoperative evaluation, intraoperative detail, and follow-up information are all covered by this system.

### Data elements

To address the objectives, we meticulously design the CRF to collect the necessary information. The CRF includes the following:

- Demographic and medical information: age, gender, height, weight, hypertension, diabetes, hyperlipidemia, ischemic stroke, coronary artery disease, renal insufficiency, chronic obstructive pulmonary disease, smoking and drinking habits, operation history, quality of life score, Rutherford grade, and ankle brachial index (ABI).
- Preoperative imaging features: The vascular condition and lesion location of the femoral popliteal artery were examined using CTA of the lower extremities artery and preoperative angiography to define the Trans-Atlantic Inter-Society Consensus II (TASC) classification and the Global Limb Anatomic Staging System. After the surgical angiography is completed, it will be assessed if the patients fit the inclusion criteria.
- Surgical information: time of operation, initial intracavitary treatment approach, lesion to be treated, lesion characteristics and degree of calcification at the treatment site, treatment plan following opening, stent graft brand, number of stent grafts, proximal and distal position of stent, treatment of thrombus, blood loss, and postoperative score of inferior knee and inferior ankle artery.
- Outcomes: Operation success rate, perioperative complications, target disease patency rate, postoperative medication management, inpatient medical expense, and secondary intervention rate.
- Follow-up: The duration of the follow-up is scheduled to be 24 months. At one, six, twelve, eighteen, and 24 months after the procedure, all of the participants will be followed up on. Quality of life score, amputation-free survival, all-cause death, non-fatal acute myocardial infarction, target vessel revascularization, ultrasonography of the treated vascular segment, ankle brachial index (ABI), current medication, smoking status, and rehospitalization are all factors to consider.

### Primary outcome measures

- Technical success rate. It is characterized as the guide wire passing neatly through the lesion. Following intervention, residual stenosis is <30% and there is no acute vascular re-occlusion 1 week following surgery (18).
- Clinically driven target lesion revascularization rate (CD-TLR) at 1, 6, 12, 18, and 24 months. Any reintervention within the target lesion due to symptoms or drop of ≥20% ABI compared to baseline.

### Secondary outcome measures

- Vascular quality of life questionnaire (VascuQol) at 1, 6, 12, 18, and 24 months. The VascuQol was designed as a questionnaire containing five domains: pain (4 items), symptoms (4 items), activities (8 items), social (2 items), and emotional (7 items) to evaluate Health related quality of life (HRQL). Every item has seven response options, with scores ranging from 1 to 7. A total score is the sum of all 25 item scores divided by 25. And both the total score as well as the domain scores range from 1 (worst HRQL) to 7 (best HRQL).The lower the value, the poorer the quality of life. Qol scores will be evaluated in relation to baseline changes.
- The rate of serious adverse events, such as myocardial infarction, ischemic stroke, cardiovascular mortality, acute limb ischemia, and amputation.
- The primary patency rate of the target lesion, as determined by color doppler ultrasonography.
- The improvement of Wound, Ischemia, and foot Infection (Wi-Fi) classification score.
- Health economics evaluation. Investigators keep track of the costs associated with the benchmark hospitalization and surgery. Through a patient survey, expenditures for further hospitalization and procedures, as well as those related to complications and adverse occurrences, are gathered. Survey questions include insurance type, the total cost and out-of-pocket expenses, length of stay, hospital level and city where the hospital is located.

### Adverse events

In a clinical study, an adverse event is any untoward medical occurrence in a patient or clinical investigation patient after providing written informed consent for participation in the study. Minor and major adverse occurrences will be classified. Minor adverse effects include problems associated with arterial puncture, distal embolism without clinical symptoms, postoperative thrombosis without severe limb ischemia, and reversible contrast-induced nephropathy. Meanwhile, major adverse events include major amputations associated with endovascular therapy, cardio- and cerebrovascular events, arterial puncture problems that require intervention, increasing hemoglobin depletion, acute renal failure, and death. In the event of major adverse effects, researchers must quickly administer appropriate therapy to the individuals. The adverse events and treatment procedure will be documented and sent to the appropriate ethical committee. Once a fatality occurs, the corresponding centers and the involved investigator shall promptly report the ethical committee with all essential information.

### Sample size and statistical analysis

This study is a prospective, multicenter, observational clinical study. We will employ a non-probabilistic sampling technique. The sample size calculation takes into account the enrollment capacity of the participating locations. Because there are a variety of endovascular therapies to treat the patients with moderate to severe femoropopliteal artery calcification in current clinical practice, so the purpose of our study is to explore the efficacy and safety of different endovascular therapies. Considering the attributes of observational design, this study can be thought as hypothesis-generating. From this perspective, the estimation of sample size only refers to feasibility, not power analysis. 600 individuals with moderate to severe calcification of the femoropopliteal artery will be recruited for this research.

Continuous variables are stated in terms of mean and standard deviation, whereas categorical variables are expressed in terms of frequency and percentage. Prior to the experiment, a normal distribution and homogeneity variance test will be conducted. For the data groups that have a normal distribution and homogeneous variances, comparisons between two groups will be made using Student's *t*-tests, and one-way ANOVA followed by Bonferroni tests for multiple groups comparisons. The global VascuQol score and each domain's changes from baseline will be handled as continuous variables and evaluated using paired *t*-tests for each time interval. The survival probability will be estimated using Kaplan-Meier estimator and the survival curve will be compared using the log-rank test or COX proportional hazard model or Weibull survival function, as appropriate. Non-parametric tests will be utilized for data groups that do not have a normal distribution or homogenous variance. In order to account for potential confounding and bias in real-world studies, a generalized linear model and multivariate analytic technique based on propensity score will be used. All statistical analyses will be performed using SPSS 26.0 software (SPSS, Chicago, IL, USA). *P*-values of < 0.05 are considered statistically significant.

## Discussion

The endovascular treatment of peripheral artery disease with calcification has remained challenging for restenosis, and available literature was limited. Restenosis has made it difficult to treat peripheral artery disease with endovascular methods, and the body of material that was accessible was sparse. Prior to the development of drug-coated or eluted technology, primary bare metal stent placement offered better patency than plain balloon angioplasty, but it also carried a higher risk of stent breakage and in-stent restenosis in lengthy infra-inguinal lesions. Drug-eluted balloons, drug-coated balloons, and stent graft have been suggested to minimize restenosis for the treatment of patients with moderate and severe calcification of the femoropopliteal artery in response to the urgent need for novel technologies. For the treatment of femoropopliteal occlusive disease, the FDA recently approved a new Drug-eluting stent called Eluvia. Different from the Zilver PTX, the stent has a layer of fluorinated polymer, which intended to optimize drug delivery over a sustained period of time. From the prospective single-arm MAJESTIC clinical trial, 24-month primary patency rates were 83.5%, 36-month free TLR was 85.3% (19, 20).

The effectiveness and safety of treatment for peripheral artery disease with calcification are still debatable despite the expanding variety of novel techniques that are currently available for endovascular therapy. Therefore, Evolution uses a study design based on actual data to close the research gap. The contemporary medical and financial landscape in health care requires attention to the economic results of clinical treatments in addition to therapeutic outcomes. The following expenses could be broken down as part of endovascular treatments: procedure costs, device costs, follow-up expenses, physician fees, and additional in-hospital expenses. Endovascular therapy prices, however, differ between nations and health systems. Furthermore, because most contemporary clinical studies have a short time frame, it is challenging to reflect mid- and long-term economic effects. Additionally, trial-based economic evaluations are unable to account for ongoing product development and frequent pricing changes. These issues highlight the need of conducting economic assessments using real-world data.

There are some limitations to the study. There will be no interference with endovascular therapies because this is an observational research. Over the 2-year follow-up period, there may be a disproportionately large percentage of individuals who are lost to follow-up.

## Conclusions

The choice of revascularization must take the patient's clinical condition, the lesion's characteristics, and the best device features into account given the rapid expansion of endovascular therapy and devices in order to assure success. The Evolution project advances knowledge of the clinical and financial effects of endovascular therapy for femoropopliteal occlusive disease with moderate and severe calcification. In the end, this information can enable future PAD clinical trials, give information to help choose the best course of treatment for patients, and improve the result for PAD patients.

## Ethics statement

The studies involving human participants were reviewed and approved by the Institutional Review Board and Human Research Ethics Committee of the First Affiliated Hospital of Zhejiang University, and by the Ethics Committees of all participating centers. The patients/participants provided their written informed consent to participate in this study.

## Author contributions

ZW, MY, and JP: conception and design. ZW and MY: administrative support. JP, LG, XF, ZF, QL, CH, HS, WS, ZS, BW, CQ, ZW, and MY: provision of study materials or patients, collection and assembly of data, manuscript writing, and final approval of manuscript. JP, BW, and CQ: data analysis and interpretation. All authors have read and agreed to the published version of the manuscript.

## Conflict of interest

The authors declare that the research was conducted in the absence of any commercial or financial relationships that could be construed as a potential conflict of interest.

## Publisher's note

All claims expressed in this article are solely those of the authors and do not necessarily represent those of their affiliated organizations, or those of the publisher, the editors and the reviewers. Any product that may be evaluated in this article, or claim that may be made by its manufacturer, is not guaranteed or endorsed by the publisher.

## Abbreviations

QOL: quality of life
CD-TLR: clinically driven target lesion revascularization rate
CRF: case report form
ABI: ankle brachial index
TASC: rans-Atlantic Inter-Society Consensus
VascuQol: Vascular quality of life questionnaire
HRQL: Health related quality of life
Wi-Fi: Wound, Ischemia, and foot Infection.
